# The association of body mass index with patient outcomes after shoulder replacement surgery: Population-based cohort study using linked national data from the United Kingdom and Denmark

**DOI:** 10.1371/journal.pmed.1004786

**Published:** 2025-11-20

**Authors:** Epaminondas Markos Valsamis, Josefine Beck Larsen, Adrian Sayers, Timothy Jones, Stephen E. Gwilym, Pia Kjær-Kristensen, Theis M. Thillemann, Inger Mechlenburg, Michael R. Whitehouse, Jonathan L. Rees

**Affiliations:** 1 Nuffield Department of Orthopaedics, Rheumatology and Musculoskeletal Sciences, Botnar Institute for Musculoskeletal Sciences, University of Oxford, Oxford, United Kingdom; 2 NIHR Oxford Biomedical Research Centre, Oxford, United Kingdom; 3 Department of Clinical Medicine, Aarhus University, Aarhus, Denmark; 4 Department of Orthopaedic Surgery, Aarhus University Hospital, Aarhus, Denmark; 5 Musculoskeletal Research Unit, Bristol Medical School, Southmead Hospital, University of Bristol, Bristol, United Kingdom; 6 Research Center for Prevention and Health Promotion, VIA University College, Aarhus, Denmark; Université Paris Cité UFR de Médecine: Universite Paris Cite UFR de Medecine, FRANCE

## Abstract

**Background:**

There is growing evidence that access to joint replacement surgery is being restricted based on body mass index (BMI) despite any formal recommendations. Our aim was to investigate the association between BMI and patient outcomes after elective primary shoulder replacement surgery to inform future commissioning and national guidance.

**Methods and findings:**

In this population-based cohort study, patients aged 18–100 years having elective primary shoulder replacement surgery were identified using linked national joint registry and hospital data from public and private hospitals in the United Kingdom (2018−22) and Denmark (2006−21). The main outcome measure was mortality within 365 days of surgery. Secondary outcome measures included mortality within 90 days, serious adverse events within 90 days, and revision surgery within 4.5 years of surgery. The association between BMI and patient outcomes was assessed using flexible parametric survival models and logistic regression models, adjusting for age, sex, deprivation, main surgical indication and American Society of Anaesthesiologists (ASA) score. 15,320 and 5,446 shoulder replacement procedures from within the United Kingdom and Denmark, respectively, met the inclusion criteria. In the United Kingdom, the average age was 72.2 years, 68.3% were female and the average BMI was 29.4 kg/m^2^. In Denmark, the average age was 70.5 years, 65.3% were female and the average BMI was 28.0 kg/m^2^. There was a decreased risk of 365-day mortality in obese (BMI 40 kg/m^2^) patients (hazard ratio (HR) 0.40 [95%CI 0.21, 0.73]) and an increased risk in underweight (BMI < 18.5 kg/m^2^) patients (HR 1.18 [95%CI 1.06, 1.32]), compared to patients with BMI 21.75 kg/m^2^. Underweight patients had an increased risk of 90-day mortality (HR 1.69 [95%CI 1.14, 2.52]), 90-day serious adverse events (odds ratio 1.36 [95%CI 1.05, 1.77]) and revision surgery (HR 1.70 [95%CI 1.25, 2.33]). Increasing BMI was not associated with a significantly increased risk of any secondary outcome. The main limitation of this study was the high proportion of missing BMI data and the small case numbers for the underweight study population (*n* = 131[UK], 70[Denmark]).

**Conclusions:**

Increasing BMI was associated with lower 365-day mortality, and no poorer outcomes after elective primary shoulder replacement surgery. This surgery is safe and effective in obese patients and access to shoulder replacements should not be restricted based on BMI alone. Clinicians and hospitals should be aware that underweight patients appear more at risk of mortality, serious adverse events and revision surgery after shoulder replacement.

## Author summary

**Why was this study done?:**

- Some healthcare commissioning groups are restricting access to elective hip and knee replacement surgery in patients with high body mass index (BMI) despite a lack of evidence
- There is no high-quality evidence for the association of BMI with patient outcomes after shoulder replacement surgery

**What did the researchers do and find?:**

- Over 20,000 procedures were analysed using linked hospital and national registry data from the United Kingdom and Denmark to investigate the association of BMI with patient outcomes after primary shoulder replacement surgery
- Patients with high BMI did not have an increased risk of mortality, serious adverse events or revision surgery after elective primary shoulder replacement surgery
- High BMI appeared to be associated with reduced mortality within 90 and 365 days of surgery
- Underweight patients were found to have an increased risk of mortality, serious adverse events and revision surgery after shoulder replacement

**What do these findings mean?:**

- Elective primary shoulder replacement surgery is safe and effective in patients with high BMI, and access to surgery should not be restricted based on BMI alone
- Underweight patients should be counselled regarding their increased risks after surgery, and more research is needed to identify the mechanisms behind this
- The main limitations of this study were the high proportion of missing BMI data and the small number of cases in the underweight study population

## Abstract 3

Epaminondas Markos Valsamis and colleagues investigate the association between BMI, patient complications and implant survival after shoulder replacement surgery using linked national datasets from the UK and Denmark.

## Introduction

Hip and knee replacements are established and effective surgical treatment options for end-stage arthritis, but in some countries like the United Kingdom, access to these procedures is often restricted based on body mass index (BMI) [1,2]. This occurs despite any formal recommendation from the National Institute for Health and Care Excellence. There is now growing evidence suggesting that this rationing is unwarranted, and that it represents a source of unjustifiable inequality in access to healthcare [3,4].

No recommendations for BMI-based access to surgery currently exist for shoulder replacements, largely due to conflicting evidence on patient outcomes [5,6]. In particular, there are heterogeneous results regarding the risk of revision in patients with high BMI, some studies reporting no effect while others report an increased risk in obese patients [7,8]. One previous small study on shoulder replacements did identify an association of BMI with early post-operative mortality, reporting that a BMI over 30 was associated with a lower risk of 90-day mortality [9]. Several studies have focussed on short-term revision risk and used BMI categorisation without adequately adjusting for confounding variables, highlighting the need for a more nuanced methodological approach.

Shoulder replacements deliver significant increases in quality of life and function in patients with shoulder arthritis [10,11]. In the context of growing international demand for shoulder replacements and high elective surgery waiting times, there is an important need for robust evidence to optimise patient outcomes while avoiding unnecessary restrictions to surgery [12,13].

The aim of this study was to investigate the association between BMI, patient complications and implant survival after elective primary shoulder replacement surgery using linked national datasets from two countries.

## Methods

### Study design and data sources

This was a population-based cohort study using routinely collected data for shoulder replacements undertaken at public and private hospitals within the United Kingdom and Denmark. In the United Kingdom, data from the National Joint Registry (NJR) was used from 1 June 2018 to 30 December 2022. In Denmark, data from the Danish Shoulder Arthroplasty Registry (DSR) was used from 1 January 2006 to 31 December 2021.

Data submission to the NJR and DSR is mandatory for all shoulder replacements occurring at public and private hospitals, and includes patient, surgeon, and operation details. Data from the NJR were used to analyse mortality and revision outcomes. Data from the DSR were used to analyse revision. The capture of primary shoulder replacements by both the NJR and DSR has been estimated to be around 95% with completeness increasing in recent years [14,15].

Data from the NJR were additionally linked to the NHS Hospital Episode Statistics Admitted Patient Care database to analyse serious adverse events (SAE) requiring admission to hospital after shoulder replacement surgery, from 1 June 2018 to 29 December 2021. The Hospital Episode Statistics Admitted Patient Care database records all inpatient and day case activities in public hospitals and publicly funded procedures in private hospitals in England, and includes demographic data, medical diagnoses, procedural and administrative information. Hospital Episode Statistics data are used to ensure accurate reimbursement of NHS providers for their activities.

Data from the DSR were linked to the Danish Anaesthesia Database, the Danish National Patient Registry and the Danish Civil Registration System for mortality and SAE analysis [16–18]. The Danish Anaesthesia Database is a national database that contained information on BMI and American Society of Anaesthesiologists (ASA) score and records activities performed by anaesthesiologists in public and private hospitals. The Danish National Patient Registry records medical diagnosis, comorbidities and administrative data on inpatient and outpatient activity and is used by healthcare authorities to reimburse Danish Hospitals. The Civil Registration System contains information on mortality and migration.

### Selection criteria

Data were available for all consenting patients aged 18–100 years having a primary shoulder replacement (including humeral hemiarthroplasty, anatomical total shoulder replacement, and reverse total shoulder replacement). Patients were included if their surgical history was consistent (e.g., date of death did not precede their surgery) and did not contain duplicates (S1 Appendix). Procedures undertaken for acute trauma or malignancy indications were excluded as they represent a distinct cohort with more variable outcomes arising from their unique presentations and associated injuries. The second of same-day bilateral primary shoulder replacements were excluded to avoid double-counting of mortality events which could lead to bias.

### Outcomes

The primary outcome was all-cause mortality at 1 year after surgery. Secondary outcomes included all-cause mortality within 90 days of surgery, SAEs within 90 days of surgery and revision surgery at 4.5 years (representing implant survival).

SAEs were defined as medical complications serious enough to require admission to hospital, including pulmonary embolism, myocardial infarction, lower respiratory tract infection, acute kidney injury, urinary tract infection, cerebrovascular events, and all-cause death [12,19]. SAEs were considered a binary event and identified using ICD-10 (International Classification of Diseases, 10th revision) codes (S1 Appendix). The NJR defines revision as a procedure that involves adding, removing, or modifying one or more components of a joint prosthesis, and is the most commonly used metric for assessing the success of joint replacement surgery [20].

### Statistical analysis

The primary exposure of interest was BMI at the time of the primary operation. This was treated as a continuous variable, and values under 15 kg/m^2^ or over 60 kg/m^2^ were considered coding errors and marked as missing. BMI started being collected by the NJR for shoulder replacements on 1 June 2018 which is the reason for choosing this start date for the NJR data. All analyses were reported using a BMI value that was centred around the average of the ‘normal weight’ category (21.75 kg/m^2^) as defined by the World Health Organisation (WHO).

Flexible parametric survival models (using restricted cubic splines to allow for modelling nonlinearity in the baseline hazard function) were used for 1-year mortality, revision, and 90-day mortality. Logistic regression was used for the binary outcome of 90-day SAEs. Models were incrementally adjusted for several covariates that were selected a priori following careful consideration and review of the existing literature. Confounding variables included age (continuous), sex (binary), index of multiple deprivation decile (categorical), main surgical indication (categorical), and ASA score (categorical: I, II, III, IV + V).

All continuous variables, including BMI, were investigated with restricted cubic splines to test for non-linearity in their association with each outcome. The continuous variable specification with the best fit as specified by the lowest Akaike Information Criterion was chosen, and visual inspection was used to investigate for overfitting.

### Sensitivity analysis

A sensitivity analysis was undertaken by additionally adjusting for procedure type as a categorical variable (humeral hemiarthroplasty; anatomical total shoulder replacement; reverse total shoulder replacement). The association of BMI with long-term revision (16.6 years) was also investigated using the Danish dataset that had longer-term follow-up available.

### Missing data

In the NJR data, index of multiple deprivation data were missing for 182 procedures (0.8%), so these were excluded, and a complete case analysis undertaken. BMI was missing in 6,724 procedures (30.5%), in keeping with completeness rates observed for hip and knee replacements in the NJR [20].

Index of multiple deprivation, a United Kingdom-specific measure, was unavailable for the Danish data. ASA was missing in 41 procedures (0.4%), so these data were excluded, and a complete case analysis undertaken. BMI was missing in 4,709 procedures (46.4%).

BMI was likely missing completely at random due to administrative errors, and therefore complete case analysis was undertaken as this would provide an unbiased estimate of the association of BMI with each outcome [3]. All analyses were undertaken using Stata v18.5 (StataCorp) [21].

### Patient and public involvement

One of the top 10 research uncertainties from the 2015 James Lind Alliance Priority Setting Partnership on shoulder surgery relates to predicting patient outcomes after surgery to support the patient-surgeon decision-making process [22] . Patient representatives sit on the committee structure of the NJR.

### Planning of analyses

The analysis plan was made prior to any analysis and this was agreed by all co-authors. No data-driven changes to the analysis plan took place. This study was reported as per the Reporting of Studies Conducted using Observational Routinely-Collected Data (RECORD) guideline (S1 Checklist).

### Ethics approval

Patient consent was obtained for data collection by the NJR. According to the specifications of the NHS Health Research Authority, separate informed consent and ethical approval were not required for the present study. In Denmark, the study was reported to the Danish Protection Agency (Journal No 1-16-02-387-22) and no ethical approval was required for the Danish data.

## Results

### Study population

The United Kingdom population comprised 22,044 elective primary shoulder replacement procedures in 20,839 patients, of which 15,320 procedures (10,466 females [68.3%], average age 72.2 years) had an available BMI score at the time of their surgery (S1 Appendix). The distribution of patient demographics including surgical information were similar between patients with and without missing BMI scores (Table 1). The average BMI was 29.4 kg/m^2^ (SD 5.7) (Table 2).

**Table 1:** Patient demographics by BMI missingness in the United Kingdom NJR data and Danish data.

	United Kingdom		Denmark	
	BMI present	BMI missing	BMI present	BMI missing
*N*	15,320 (69.5%)	6,714 (30.5%)	5,446 (53.6%)	4,709 (46.4%)
Age/years (SD)	72.2 (9.5)	72.2 (9.9)	70.5 (10.2)	69.4 (10.5)
Sex				
Male	4,854 (31.7%)	2,173 (32.4%)	1,889 (34.7%)	1,831 (38.9%)
Female	10,466 (68.3%)	4,541 (67.6%)	3,557 (65.3%)	2,878 (61.1%)
ASA category				
1	907 (5.9%)	336 (5.0%)	653 (12.0%)	27 (0.6%)
2	9,673 (63.1%)	4,129 (61.5%)	3,395 (62.3%)	112 (2.4%)
3	4,644 (30.3%)	2,193 (32.7%)	1,368 (25.1%)	50 (1.1%)^*^
4 and 5	96 (0.6%)	56 (0.8%)	30 (0.6%)	
Missing	0 (0.0%)	0 (0.0%)	0 (0.0%)	4,520 (96.0%)
Primary surgical indication				
AVN	351 (2.3%)	219 (3.3%)	258 (4.7%)	271 (5.8%)
CTA	4,041 (26.4%)	1,753 (26.1%)	1,472 (27.0%)	1,141 (24.2%)
Inflammatory	482 (3.1%)	210 (3.1%)	238 (4.4%)	210 (4.5%)
OA	8,710 (56.9%)	3,567 (53.1%)	2,792 (51.3%)	2,471 (52.5%)
Other	749 (4.9%)	376 (5.6%)	49 (0.9%)	83 (1.8%)
Trauma sequelae	987 (6.4%)	589 (8.8%)	637 (11.7%)	533 (11.3%)
IMD decile				
1 (most deprived)	1,345 (8.8%)	698 (10.4%)		
2	924 (6.0%)	415 (6.2%)		
3	1,009 (6.6%)	490 (7.3%)		
4	1,303 (8.5%)	585 (8.7%)		
5	1,549 (10.1%)	615 (9.2%)		
6	1,859 (12.1%)	720 (10.7%)		
7	1,883 (12.3%)	778 (11.6%)		
8	1,771 (11.6%)	760 (11.3%)		
9	1,904 (12.4%)	786 (11.7%)		
10 (least deprived)	1,773 (11.6%)	867 (12.9%)		
Procedure type				
HA	830 (5.4%)	462 (6.9%)	1,346 (24.7%)	1,661 (35.3%)
RTSR	9,817 (64.1%)	4,534 (67.5%)	2,427 (44.6%)	1,495 (31.7%)
TSR	4,673 (30.5%)	1,718 (25.6%)	1,673 (30.7%)	1,553 (33.0%)

Values reported as frequencies (percentage) unless otherwise indicated. ASA, American Society of Anaesthesiologists. AVN, Avascular necrosis; CTA, Cuff tear arthropathy; OA, Osteoarthritis; HA, humeral hemiarthroplasty; RTSR, Reverse total shoulder replacement; TSR, anatomical total shoulder replacement; IMD, Index of Multiple Deprivation (United Kingdom-specific variable).

* Small samples less than three were suppressed in line with Statistics Denmark’s requirements, so a combined category of ASA 3/4/5 was reported.

**Table 2:** BMI distribution of study population using World Health Organisation (WHO) categories.

BMI category	Nutritional status	Number (percentage)	
		United Kingdom	Denmark
<18.5	Underweight	131 (0.9%)	70 (1.3%)
18.5–25	Normal weight	2,822 (18.4%)	1,533 (28.1%)
25–30	Pre-obesity	5,618 (36.7%)	2,040 (37.5%)
30–35	Obesity class I	4,081 (26.6%)	1,146 (21.0%)
35–40	Obesity class II	1,857 (12.1%)	509 (9.3%)
>40	Obesity class III	811 (5.3%)	148 (2.7%)

Values reported as frequencies (percentage) unless otherwise indicated. Underweight: <18.5 kg/m^2^, Normal weight: 18.5–24.9 kg/m^2^, Pre-obesity: 25.0–29.9 kg/m^2^, Obesity class I: 30.0–34.9 kg/m^2^, Obesity class II: 35.0–39.9 kg/m^2^, Obesity class III: >40 kg/m^2^.

The Danish population comprised 10,155 elective primary shoulder replacement procedures in 8,976 patients, of which 5,446 procedures (3,557 females (65.3%), average age 70.5 years) had an available BMI and ASA score at the time of their surgery (Table 1). The average BMI was 28.0 kg/m^2^ (SD 5.5).

For the analysis of 90-day SAEs that used linked data from the NJR and NHS Hospital Episode Statistics, the study population comprised 13,630 elective primary shoulder replacement procedures in 13,034 patients, of which 9,304 procedures (6,400 females [68.8%], average age 72.3 years) had an available BMI score (average BMI was 29.6 kg/m^2^ (SD 5.8)) at the time of their surgery (S1 Appendix). For data from the United Kingdom, patients with a higher BMI had a higher Charlson Comorbidity Index based on all their preoperative past medical history, while this was not observed in data from Denmark (S1 Appendix) [23].

### Primary outcome: Mortality within 365 days

**Fig 1:**
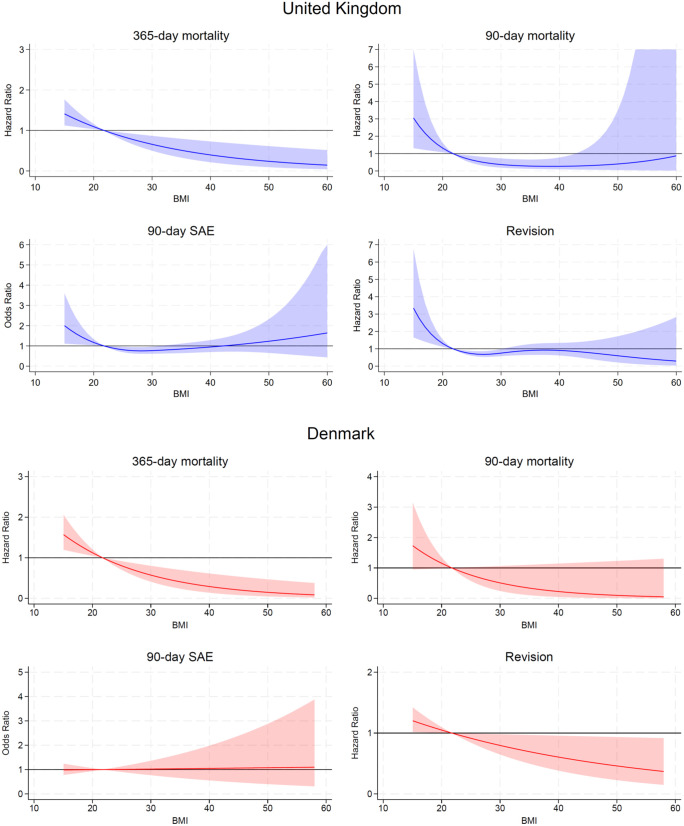
Association of BMI with primary and secondary outcomes after adjusting for age, sex, index of multiple deprivation (for the United Kingdom data), primary surgical indication and American Association of Anaesthesiologists (ASA) score. Reference (hazard ratio of 1) of BMI 21.75 kg/m^2^ (average of ‘normal weight’ World Health Organisation (WHO) category). Shaded areas represent 95% confidence intervals.

For the United Kingdom data, there were 136 deaths within 365 days of primary surgery with a total of 4,891,183 days of observation time. In the adjusted analysis (age, sex, index of multiple deprivation decile (for the United Kingdom data), primary surgical indication and ASA score), a higher BMI was found to be associated with a reduced hazard ratio (HR) of 365-day mortality (Fig 1). Compared to the reference BMI of 21.75 kg/m^2^, patients with a lower BMI had an increased HR of 365-day mortality, with patients of BMI 18.5 kg/m^2^ having a HR of 1.18 (95% CI 1.06, 1.32). Those with a higher BMI had a decreased risk, with patients of BMI 40 kg/m^2^ having a HR of 0.40 (95% CI 0.21, 0.73). For the Danish data, there were 101 deaths within 365 days of primary surgery with a total of 1,969,306 days of observation time, and a very similar association to that seen with the United Kingdom data was observed (Table 3).

**Table 3:** Association of BMI with primary and secondary outcomes after adjusting for age, sex, index of multiple deprivation (for the United Kingdom data), primary surgical indication and American Association of Anaesthesiologists (ASA) score.

Country	BMI	365-day mortality			90-day mortality			90-day SAE			Revision		
		HR	LCI	UCI	HR	LCI	UCI	OR	LCI	UCI	HR	LCI	UCI
United Kingdom	18.5	1.180	1.058	1.317	1.691	1.140	2.519	1.363	1.052	1.769	1.703	1.248	2.332
	25	0.848	0.760	0.945	0.629	0.451	0.876	0.810	0.686	0.956	0.720	0.593	0.875
	30	0.657	0.498	0.867	0.368	0.187	0.724	0.767	0.612	0.962	0.752	0.573	0.986
	35	0.510	0.327	0.795	0.279	0.119	0.657	0.845	0.642	1.112	0.906	0.646	1.271
	40	0.395	0.214	0.729	0.266	0.092	0.774	0.946	0.695	1.288	0.906	0.613	1.340
Denmark	18.5	1.246	1.091	1.423	1.306	0.976	1.747	0.992	0.885	1.111	1.094	1.008	1.187
	25	0.803	0.704	0.917	0.766	0.573	1.024	1.008	0.900	1.129	0.914	0.843	0.992
	30	0.573	0.410	0.802	0.509	0.244	1.062	1.021	0.766	1.362	0.797	0.648	0.980
	35	0.408	0.238	0.700	0.337	0.103	1.102	1.035	0.651	1.644	0.694	0.497	0.969
	40	0.292	0.139	0.613	0.224	0.044	1.143	1.048	0.555	1.980	0.605	0.383	0.957

Reference (hazard ratio of 1) of BMI 21.75 kg/m^2^ (average of ‘normal weight’ World Health Organisation (WHO) category). HR, hazard ratio; OR, odds ratio; LCI, lower confidence interval; UCI, upper confidence interval; SAE, serious adverse events.

### Secondary outcomes: Mortality within 90 days, serious adverse events within 90 days and revision surgery

For the United Kingdom data, there were 33 deaths within 90 days of primary surgery with a total of 1,341,484 days of observation time. In the adjusted analysis, a higher BMI was found to be associated with a reduced HR of 90-day mortality up to a BMI of around 40 kg/m^2^ (Fig 1, Table 3). Thereafter, this association was attenuated back towards null, and this was not significantly different compared to the reference BMI as the confidence intervals cross one. Compared to the reference BMI of 21.75 kg/m^2^, patients with a lower BMI had an increased risk of 90-day mortality, with patients of BMI 18.5 kg/m^2^ having a HR of 1.69 (95% CI 1.14, 2.52). Those with a higher BMI had a decreased risk, with patients of BMI 40 kg/m^2^ having a HR of 0.27 (95% CI 0.09, 0.77). For the Danish data, there were 22 deaths within 90 days of primary surgery with a total of 489,146 days of observation time. A similar estimated association to that seen in the United Kingdom data was observed, although this was not significant due to the confidence intervals crossing one.

For the United Kingdom data, there were 437 SAEs (4.70%) within 90 days of primary surgery. In the adjusted analysis, a low BMI was associated with an increased risk of 90-day SAEs. This risk decreased with increasing BMI, up to around 28 kg/m^2^, while a high BMI did not show a significantly different risk. Compared to the reference BMI of 21.75 kg/m^2^, patients with BMI 18.5 kg/m^2^ had an odds ratio (OR) of 1.36 (95% CI 1.05, 1.77), while patients with BMI 30 kg/m^2^ had an OR of 0.77 (95% CI 0.61,0.96). For the Danish data, there were 320 SAEs (5.88%) within 90 days of primary surgery, and no significant association was observed.

For the United Kingdom data, there were 261 revisions with a maximum follow-up of 4.58 years and 34,812.69 years of observation time. In the adjusted analysis, a higher BMI was found to be associated with a decreased HR of revision up to a BMI of around 27 kg/m^2^, and there was no significantly different risk above 30 kg/m^2^. Compared to the reference BMI of 21.75 kg/m^2^, patients with BMI 18.5 kg/m^2^ had a HR of 1.70 (95% CI 1.25, 2.33), while patients with BMI 30 kg/m^2^ had a HR of 0.75 (95% CI 0.57, 0.99). For the Danish data, there were 229 revisions with a maximum follow-up of 4.58 years (matching the United Kingdom data) and 23,033.253 years of observation time. A similar association to that seen in the United Kingdom data was observed, though the increased risk of revision at low BMI was smaller in magnitude, and the decreased risk of revision with a higher BMI continued for high BMI values above 50 kg/m^2^.

### Sensitivity analysis

Incrementally adjusting for a different number of confounding variables had little effect on the observed association of BMI with any outcome (S1 Appendix). Additionally adjusting for procedure type had a negligible effect on all associations. For the Danish data, a similar association was identified for the association of BMI with long-term (16.6-year) revision risk (S1 Appendix).

## Discussion

This study used national, linked, routinely collected registry and hospital data from both the United Kingdom and Denmark to investigate the association of patient BMI with mortality, SAEs and revision surgery after elective primary shoulder replacement surgery. Increased BMI was found to be associated with a lower risk of mortality within 365 days of primary surgery. Patients with low BMI appeared to be at a higher risk of 90-day mortality, 90-day SAEs and revision surgery in the United Kingdom. Patients with high BMI did not have a significantly higher risk of any complication when compared to patients at the average of the ‘normal weight’ category. The results from the two countries were notably consistent, supporting the robustness of the results; observed differences may be explained by variations in sample size, healthcare systems, and the characteristics of patients undergoing surgery.

The main strengths of this study lie in its large sample size and robust methods. Firstly, we investigated the association of BMI with multiple patient outcomes, made possible by using linked national registry and hospital data. Secondly, the data used reflect all surgery undertaken across different geographic regions, representing all the main types of shoulder replacement procedures and patients of different ages, ethnicity, and socioeconomic groups, providing a complete picture of shoulder replacement activity across the United Kingdom and Denmark for elective primary shoulder replacement performed for a variety of indications. Thirdly, the use of restricted cubic splines to model both age and BMI avoided any loss of information from categorisation and allowed for the non-linear relationship of BMI with each outcome to be delineated. Finally, extensive confounding adjustment transparently demonstrated the consistent association of BMI with each outcome measure, including when additionally adjusting for procedure type in a sensitivity analysis.

Despite these strengths, the study has certain limitations. Firstly, the proportion of missing data in BMI is considerable (30.5% in the United Kingdom and 46.4% in Denmark). However, patient demographics were similar between procedures with missing and non-missing BMI data, suggesting it is unlikely for there to be responder bias. Second, it is possible that obese patients receiving surgery were healthier and fitter than similar people not having surgery due to a healthy patient selection effective in elective surgical pathways which may have resulted in selection bias. However, obese patients did have a greater number of medical comorbidities than non-obese patients in the United Kingdom data, and the association of obesity with reduced mortality persisted despite adjusting for ASA score. Thirdly, it is possible that patients requiring revision surgery may have been deemed unsuitable because of increased comorbidities or a high BMI, and as such, display a falsely reduced revision risk. The incompleteness of patient-reported outcome data collected by the NJR for shoulder replacement surgery precludes meaningful analysis that could clarify whether more obese patients have different patient-reported outcomes. Fourthly, while our results represent similar trends in two different countries, the results may not be generalisable to other countries and healthcare systems. Fifthly, the small number of events at the extremes of BMI, particularly for the rarest outcomes like 90-day mortality, increases the uncertainty of the observed association, and this is reflected in the wide confidence intervals at these extremes. This includes both underweight as well as very high BMI patients. Finally, the study used observational data and as such, causality cannot be inferred.

The ‘obesity paradox’ has previously been identified in hip and knee replacements, where obesity appears to be associated with reduced early mortality after surgery [3,24]. However, the association of high BMI with reduced mortality demonstrated in our study on shoulder replacements is even greater than that identified for hips and knees. A similar obesity paradox has also been observed for surgeries across different specialties, including vascular and cardiac surgery [25,26]. Possible explanations for this include increased lean body mass, protective peripheral body fat and a reduced inflammatory response [27]. Chronic diseases and surgery are characterised by increased inflammatory responses, and adipose tissue is known to regulate inflammation by secreting TNA-α receptors and adipokines [28,29]. This, together with genetic theories, offer potential explanations for the obesity paradox.

Previous studies investigating the association of BMI with patient outcomes have reported heterogeneous results, and many have considerable limitations, including revision being considered a binary outcome as opposed to a time-to-event outcome (resulting in bias from censoring), failing to account for non-linearity in continuous variables or adjusting for a limited number of confounding variables [6–9]. One possible explanation for the lack of an increased revision risk in patients with high BMI, is that the shoulder is not a weight-bearing joint like lower limb joints and therefore may not be subjected to the increased mechanical stress that can result in implant loosening observed with hip and knee replacements. Low BMI, with associated osteopenia and subsequent implant loosening could be a possible explanation for the observed increased revision risk in underweight patients or there may be factors associated with low BMI that are not captured in the data available for analysis. There is evidence that underweight people have a higher baseline risk of mortality; it is uncertain, however, whether the increased risk observed in our study reflects an additional excess beyond this baseline [30]. Other studies also identified no increased risk of postoperative medical complications with increased BMI [7].

The results of this study are important for patients, surgeons and healthcare policymakers, as they identify that obese patients deemed fit enough for surgery do not have poorer outcomes after primary shoulder replacement surgery, as has been previously suggested. In fact, obesity in those patients undergoing shoulder replacement surgery appears to be associated with a reduced risk of 365-day mortality and is not associated with increased risk of revision surgery by 4.5 years. Contrastingly, our data suggests that, at least in the United Kingdom, underweight patients were found to be at an increased risk of 90-day and 365-day mortality, as well as 90-day medical complications and revision surgery when compared to patients of ‘normal’ weight.

This is particularly relevant given that some NHS Integrated Care Systems (previously Clinical Commissioning Groups) in the United Kingdom have restricted patient access to hip and knee replacement surgery by setting BMI thresholds despite poor evidence to support this. Similarly, private healthcare providers conducting NHS joint replacement waiting list initiatives in some parts of the UK also restrict access on BMI thresholds. It is important that these policies are not transferred to shoulder replacement surgery when the evidence presented here does not support it. On the contrary, clinicians and hospitals should be encouraged to ensure that underweight patients are adequately counselled regarding their increased risks.

Further research is required to identify the mechanisms behind the observed obesity paradox, including whether BMI is associated with different indications for revision surgery, and to confirm that there is no secular selection bias that could confound these findings. Analysis of patient-reported outcomes can also elucidate whether patients with poorer outcomes are being denied revision surgery on the basis of their BMI. More research is also needed to better understand the reason why underweight patients may be experiencing worse outcomes, and to assess whether their risk of mortality is elevated beyond the baseline risk inherent to this population.

## Supporting information

S1 Appendix Supplementary material. ICD-10 codes, Data flowcharts, Incremental model and sensitivity model results, Charlson Comorbidity Index by BMI.
(DOCX)

S1 Checklist STROBE and RECORD checklist. RECORD, Reporting of studies Conducted using Observational Routinely-collected Data; For more information please see: https://www.record-statement.org/. STROBE, Strengthening the Reporting of Observational Studies in Epidemiology. For more information please see: https://www.strobe-statement.org/.
(PDF)

## Abbreviations

ASA: American Society of Anaesthesiologists
BMI: body mass index
DSR: Danish Shoulder Arthroplasty Registry
HR: hazard ratio
NJR: National Joint Registry
OR: odds ratio
RECORD: Reporting of Studies Conducted using Observational Routinely-Collected Data
SAE: serious adverse events
WHO: World Health Organisation
