# Supplementary material for: The association of body mass index with patient outcomes after shoulder replacement surgery: Population-based cohort study using linked national data from the United Kingdom and Denmark
Source: PLoS Med. 2025 Nov 20;22(11):e1004786. doi: 10.1371/journal.pmed.1004786 (PMC12633913; doi:10.1371/journal.pmed.1004786)

**Supplementary material**

[ICD-10 codes for serious adverse events 3](#_Toc194698345)

[Data 4](#_Toc194698346)

[United Kingdom National Joint Registry (NJR) data flowchart 4](#_Toc194698347)

[United Kingdom linked NJR-NHS Hospital Episode Statistics data flowchart 5](#_Toc194698348)

[Denmark linked DSR-DNPR-CPR data flowchart 6](#_Toc194698349)

[United Kingdom linked NJR and NHS Hospital Episode Statistics data demographics 7](#_Toc194698350)

[Incremental models and sensitivity analyses 8](#_Toc194698351)

[Association of BMI with 365-day mortality 8](#_Toc194698352)

[Association of BMI with 90-day mortality 9](#_Toc194698353)

[9](#_Toc194698354)

[Association of BMI with 90-day SAE 10](#_Toc194698355)

[Association of BMI with revision 11](#_Toc194698356)

[Association of BMI with long term revision (Denmark) 12](#_Toc194698357)

[12](#_Toc194698358)

[Charlson Comorbidity Index by BMI 13](#_Toc194698359)

ICD-10 codes for serious adverse events

| **Event** | **ICD-10 codes** |
| --- | --- |
| Pulmonary embolism | I26 |
| Myocardial infarction | I21,I22 |
| Cerebrovascular event | I60,I61,I62,I63,I64 |
| Acute kidney injury | N17 |
| Lower respiratory tract infection | J12,J13,J14,J15,J16,J18,J22,J86,J440,J851,J690 |
| Urinary tract infection | N10,N300,N308,N309,N390 |
| Death | *Mortality data* |

Data

United Kingdom National Joint Registry (NJR) data flowchart

Flowchart for unlinked National Joint Registry (NJR) dataset used for mortality and revision outcomes analysis


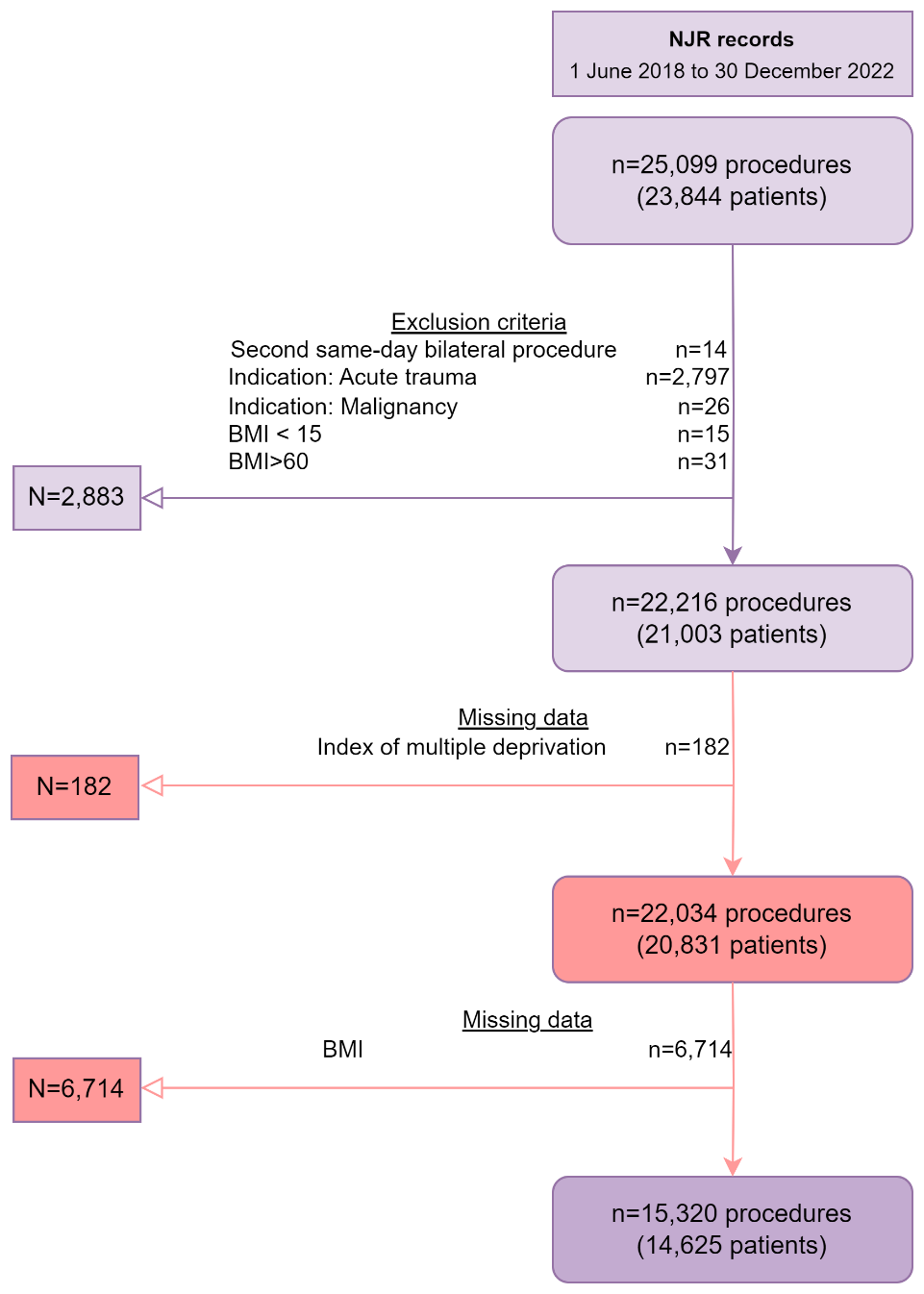


United Kingdom linked NJR-NHS Hospital Episode Statistics data flowchart

Flowchart for linked NJR and NHS Hospital Episode Statistics (HES) dataset used for serious adverse events analysis


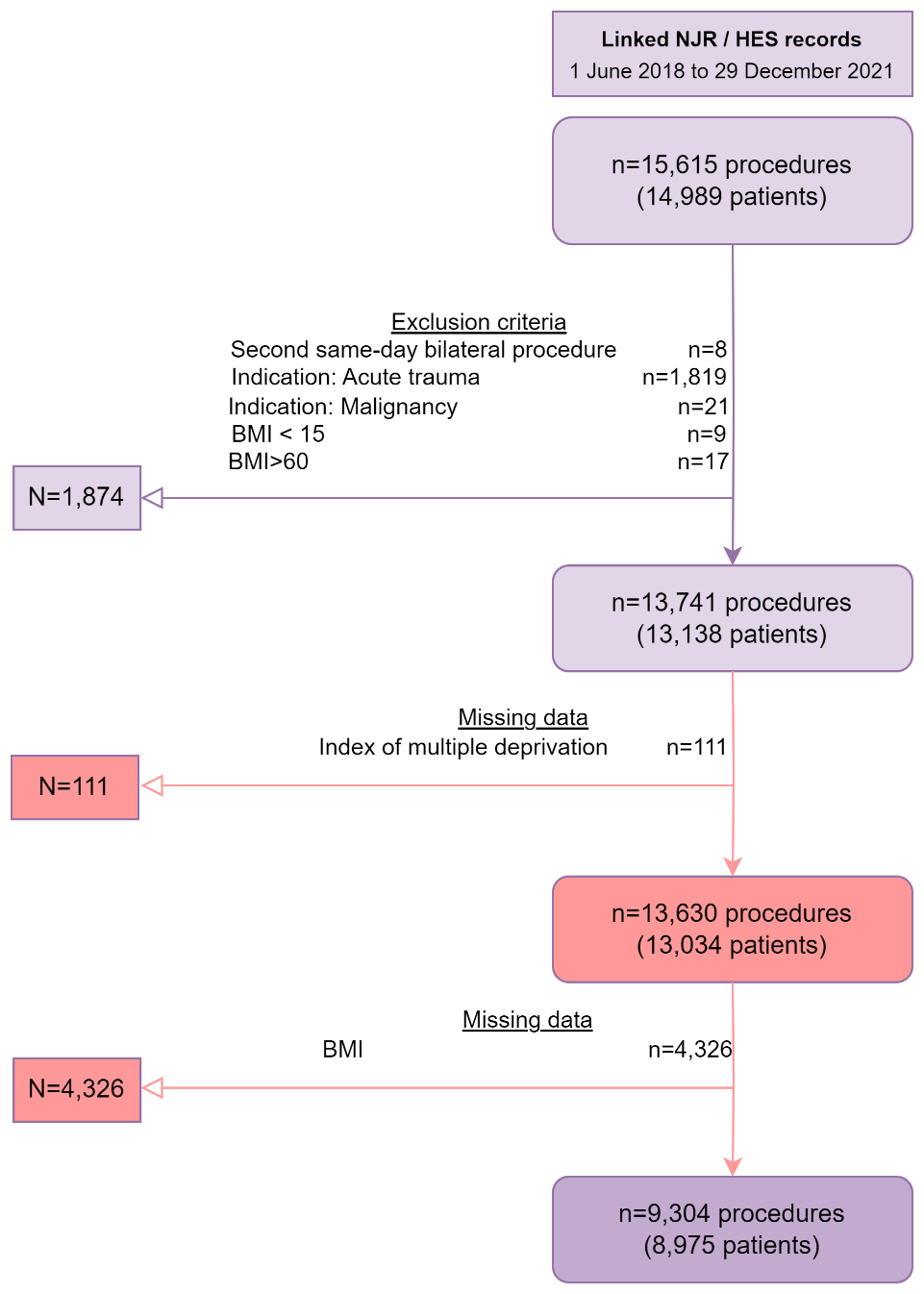


Denmark linked DSR-DAD-DNPR-CPR data flowchart

Flowchart for linked Danish Shoulder Arthroplasty Registry (DSR), Danish National Patient Registry (DNPR), Danish Anaesthesia Database (DAD), and Civil Registration System (CPR) dataset used for mortality, revision and serious adverse events analysis.


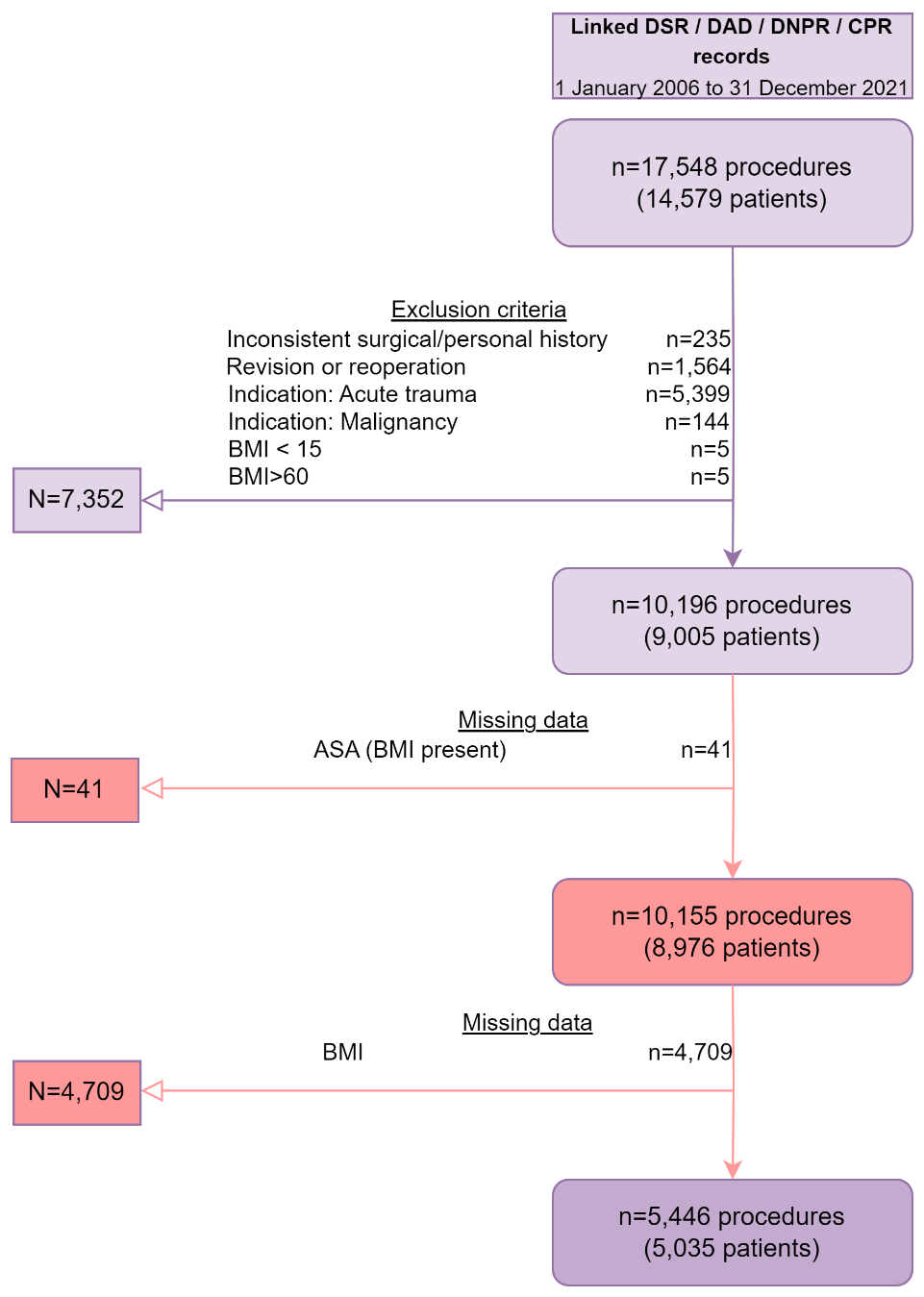


United Kingdom linked NJR and NHS Hospital Episode Statistics data demographics

*Patient demographics by BMI missingness for United Kingdom linked NJR and NHS Hospital Episodes Statistics data used for 90-day serious adverse events (SAE). ASA, American Society of Anaesthesiologists. AVN, Avascular necrosis. CTA, Cuff tear arthropathy. OA, Osteoarthritis. HA, humeral hemiarthroplasty. RTSR, Reverse total shoulder replacement. TSR, anatomical total shoulder replacement. IMD, Index of Multiple Deprivation.*

|  | BMI present | BMI missing |
| --- | --- | --- |
| N | 9,304 (68.3%) | 4,326 (31.7%) |
| Age | 72.3 (9.3) | 72.4 (9.8) |
| Sex |  |  |
| Male | 2,904 (31.2%) | 1,356 (31.3%) |
| Female | 6,400 (68.8%) | 2,970 (68.7%) |
| ASA category |  |  |
| 1 | 498 (5.4%) | 216 (5.0%) |
| 2 | 5,822 (62.6%) | 2,637 (61.0%) |
| 3 | 2,910 (31.3%) | 1,441 (33.3%) |
| 4 & 5 | 74 (0.8%) | 32 (0.7%) |
| Primary surgical indication | |  |
| AVN | 221 (2.4%) | 139 (3.2%) |
| CTA | 2,587 (27.8%) | 1,165 (26.9%) |
| Inflammatory | 307 (3.3%) | 137 (3.2%) |
| OA | 5,105 (54.9%) | 2,242 (51.8%) |
| Other | 463 (5.0%) | 252 (5.8%) |
| Sequelae | 621 (6.7%) | 391 (9.0%) |
| IMD decile |  |  |
| 1 | 650 (7.0%) | 309 (7.1%) |
| 2 | 650 (7.0%) | 314 (7.3%) |
| 3 | 667 (7.2%) | 350 (8.1%) |
| 4 | 868 (9.3%) | 404 (9.3%) |
| 5 | 1,004 (10.8%) | 418 (9.7%) |
| 6 | 1,182 (12.7%) | 478 (11.0%) |
| 7 | 1,150 (12.4%) | 511 (11.8%) |
| 8 | 1,067 (11.5%) | 512 (11.8%) |
| 9 | 1,094 (11.8%) | 495 (11.4%) |
| 10 | 972 (10.4%) | 535 (12.4%) |
| Procedure type |  |  |
| HA | 517 (5.6%) | 322 (7.4%) |
| RTSR | 6,026 (64.8%) | 2,884 (66.7%) |
| TSR | 2,761 (29.7%) | 1,120 (25.9%) |

Incremental models and sensitivity analyses

Association of BMI with 365-day mortality


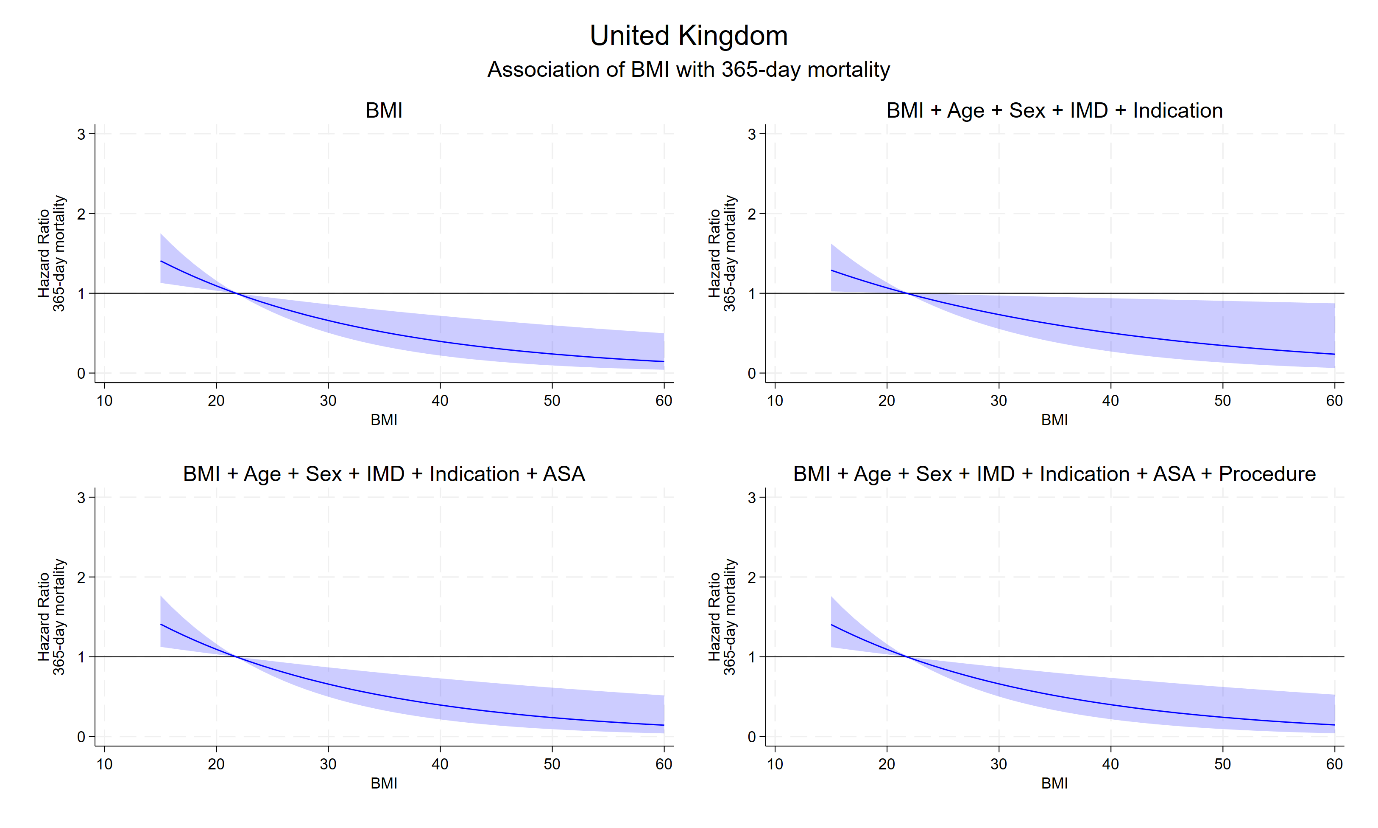


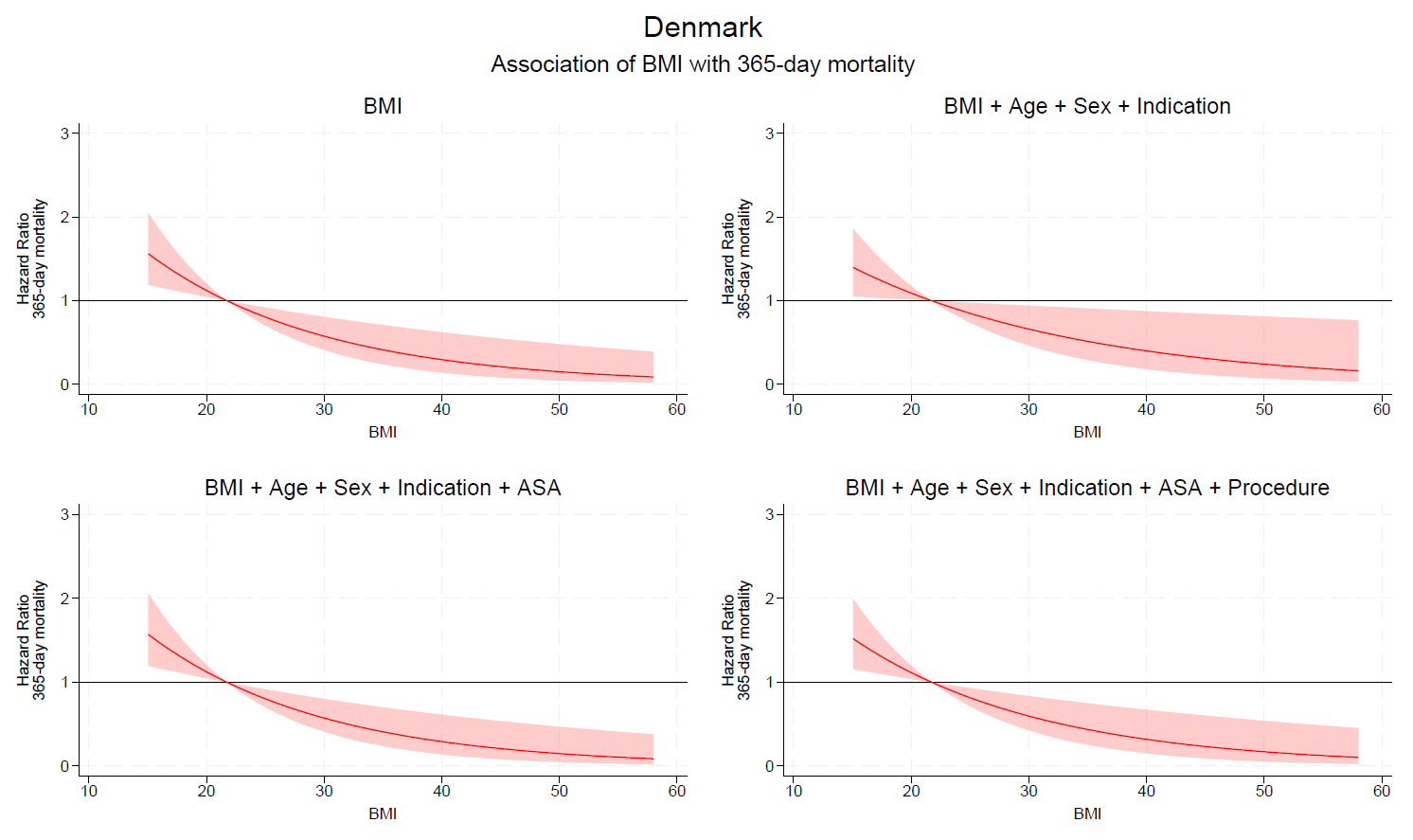


Association of BMI with 90-day mortality


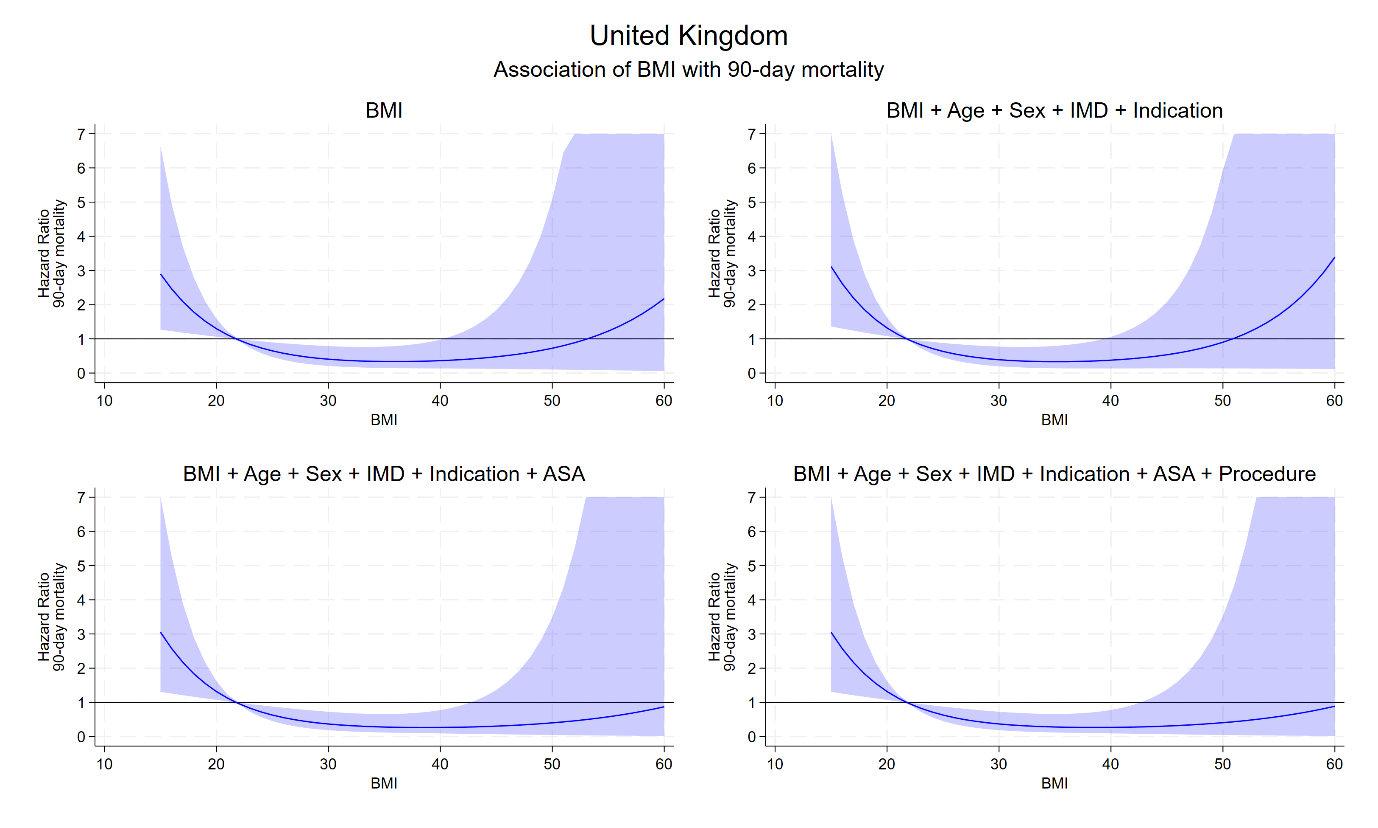


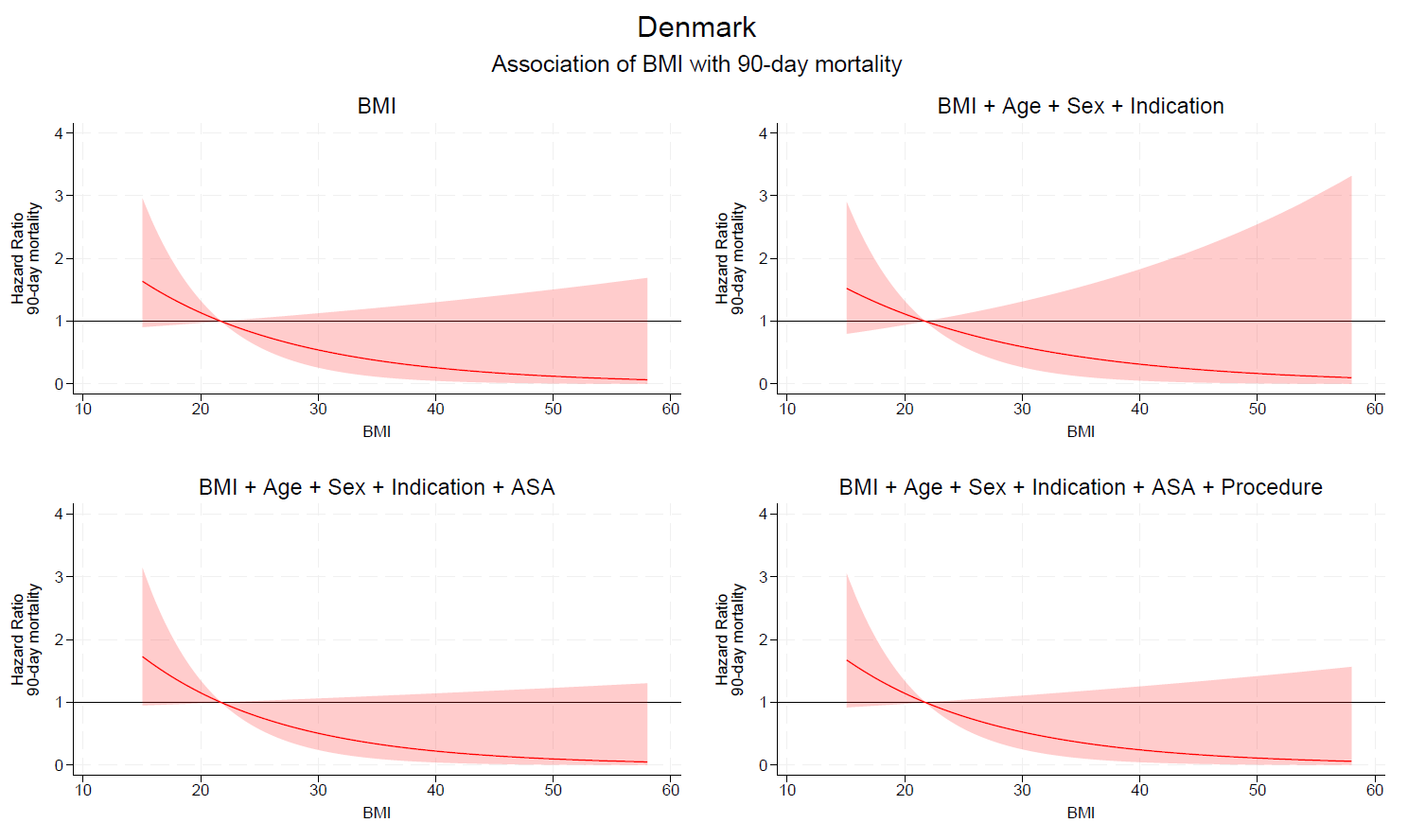


Association of BMI with 90-day SAE


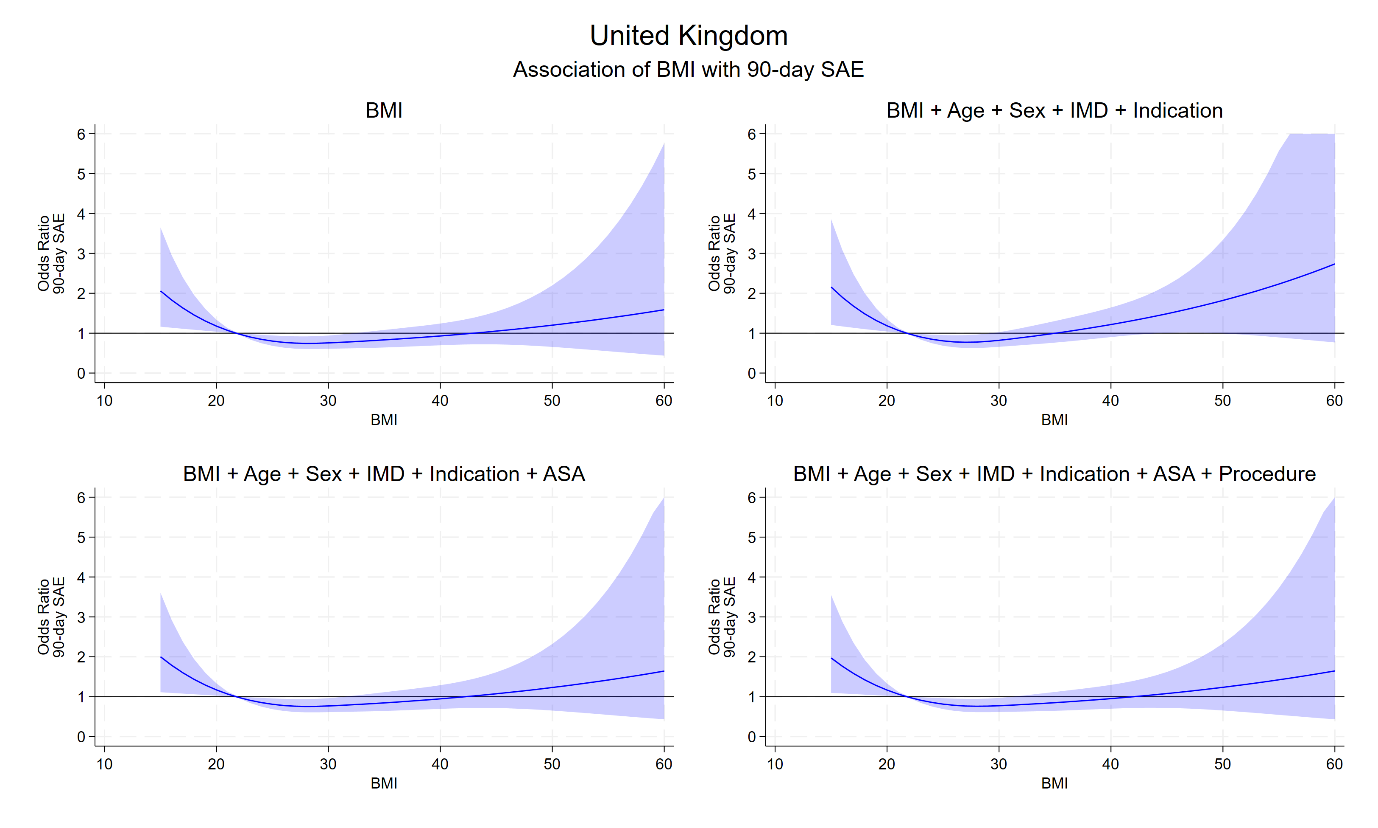


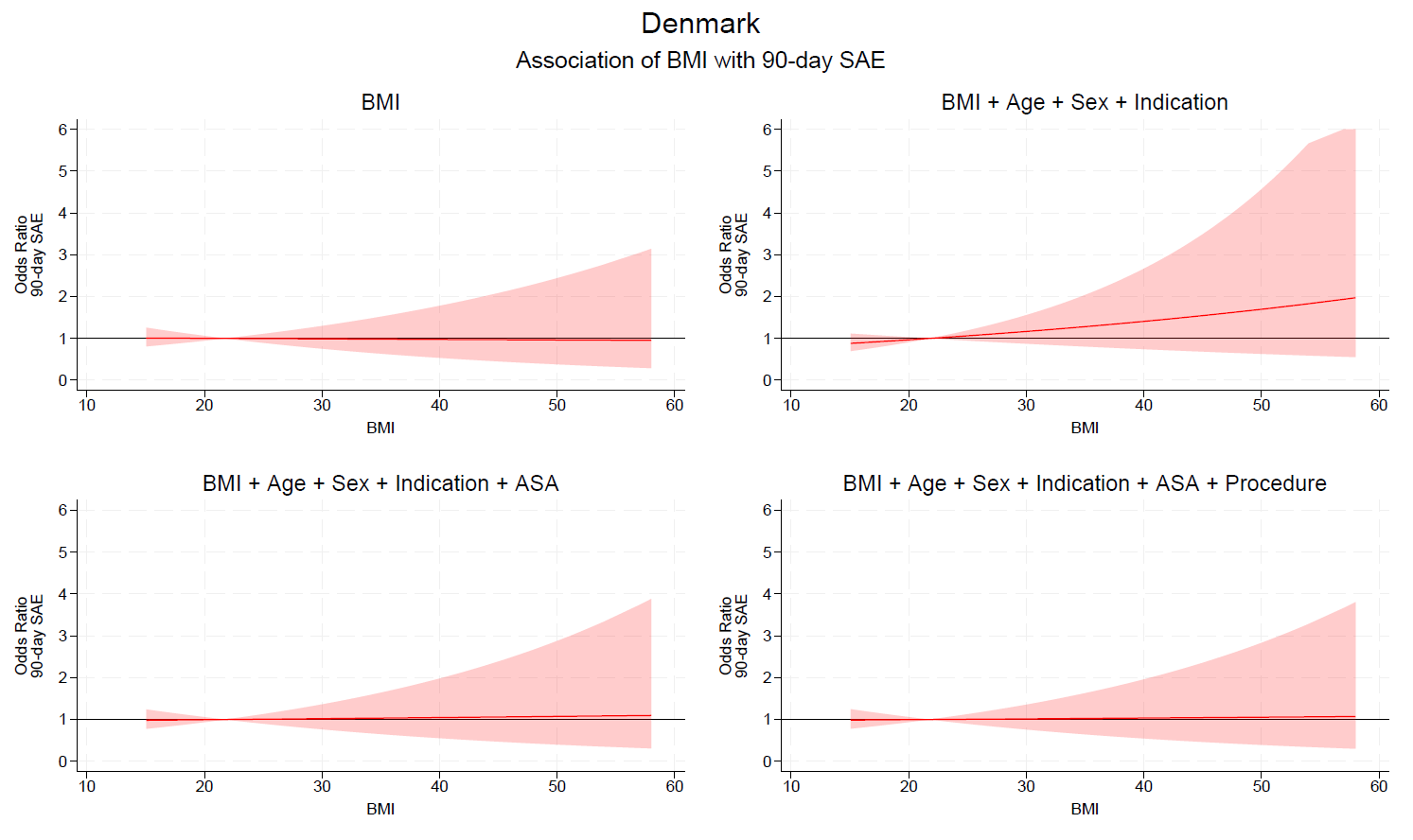


Association of BMI with revision


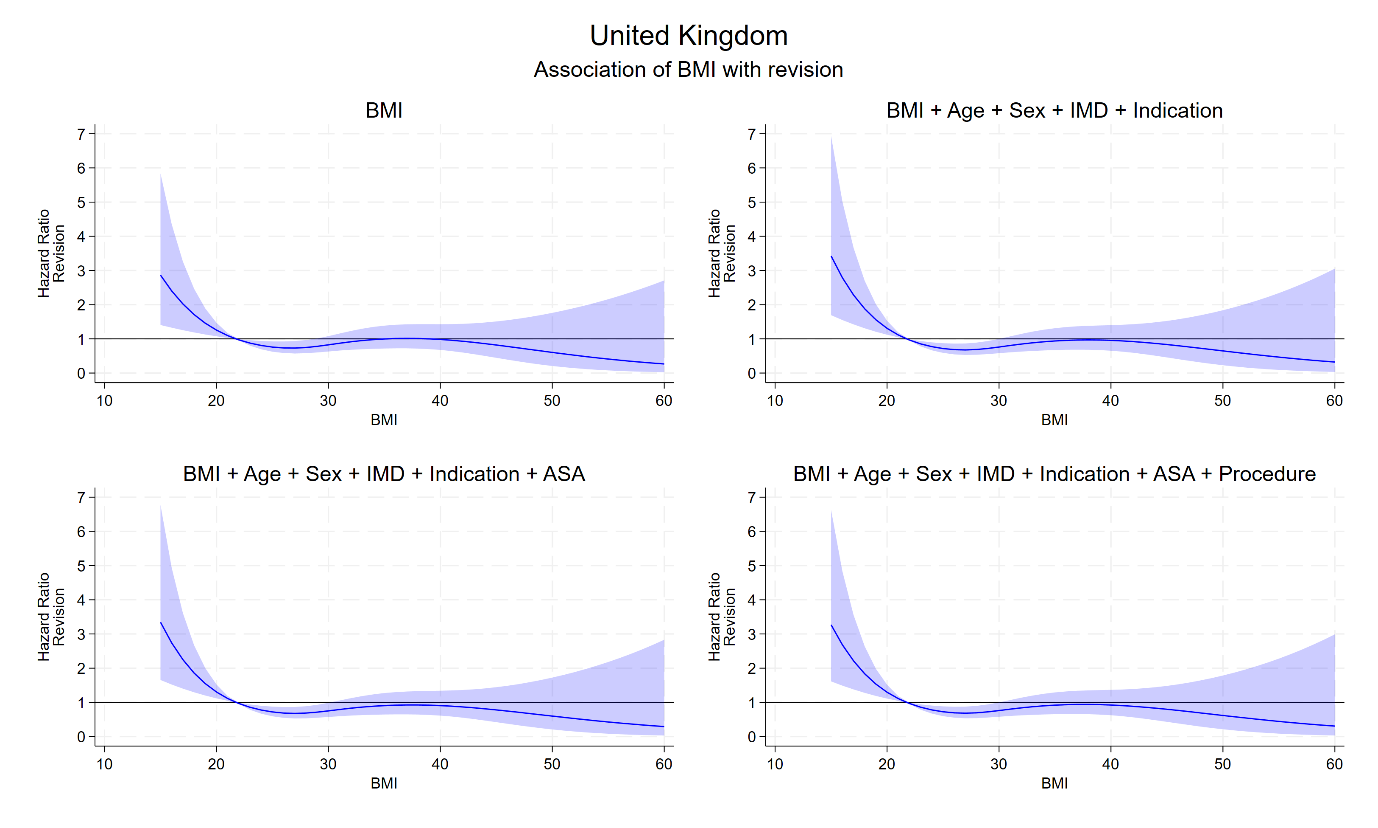


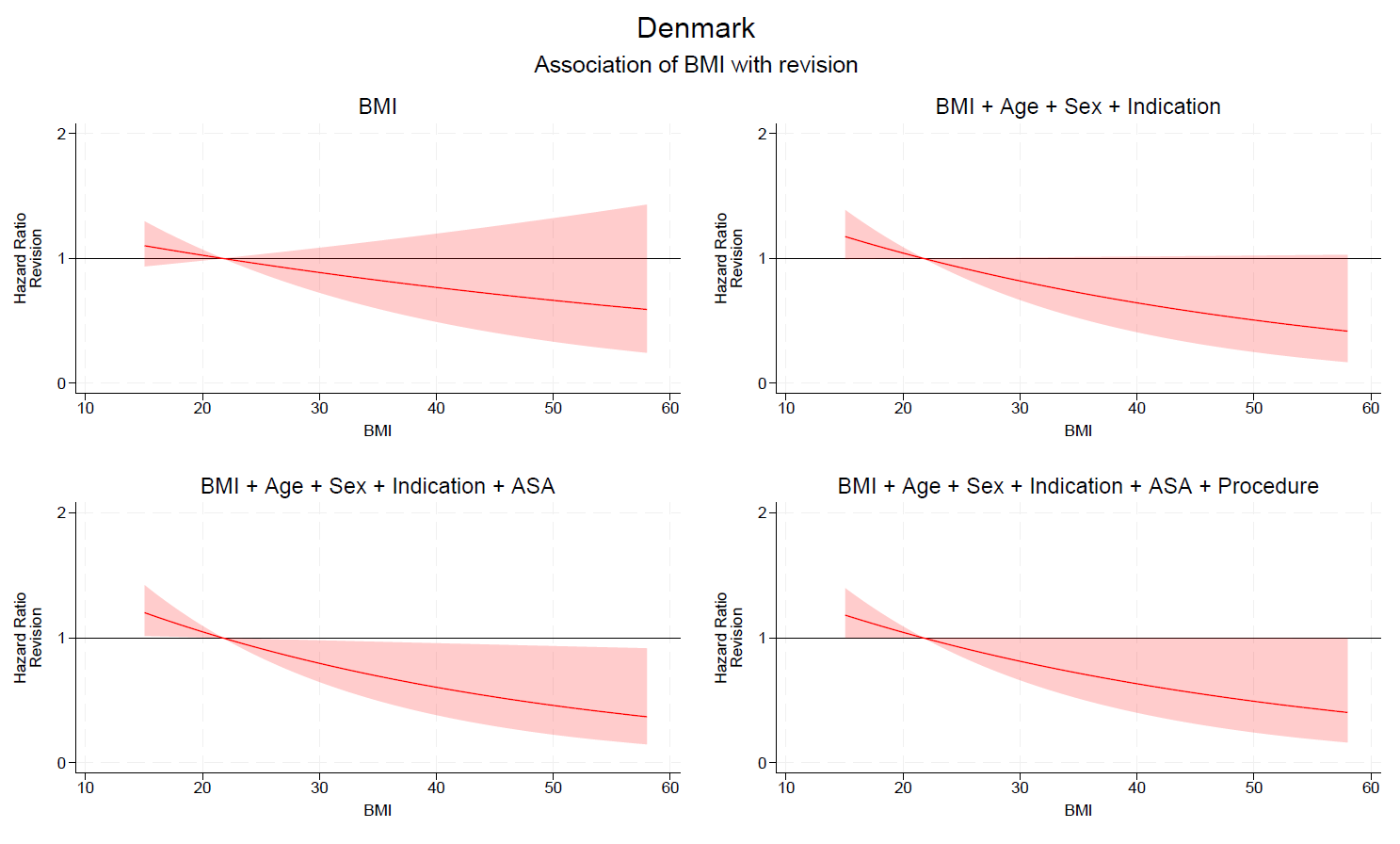


Association of BMI with long term revision (Denmark)

There were 320 revisions with a maximum follow-up of 16.6 years and 25,980.63 years of observation time.


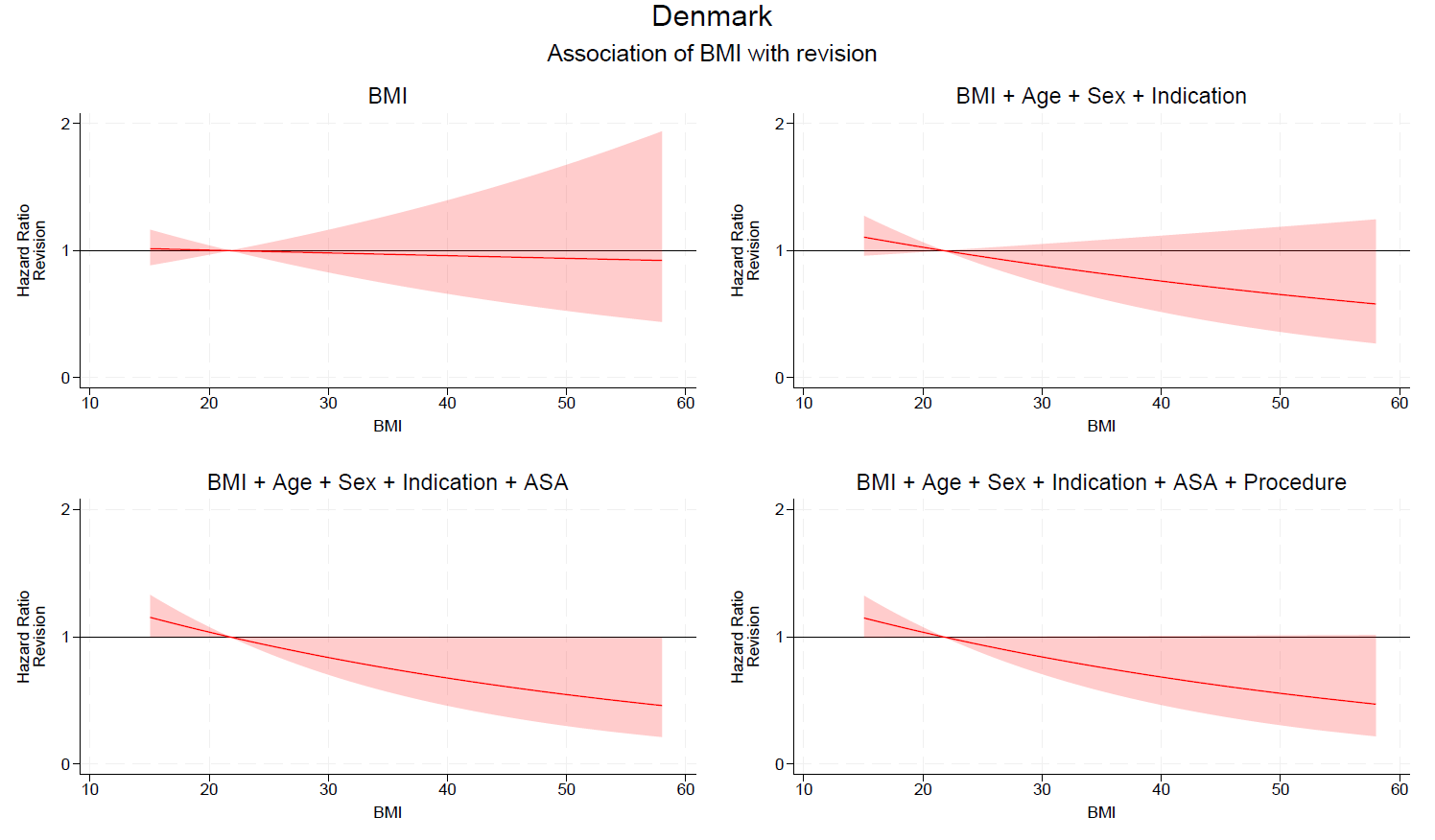


The table below shows the hazard ratio of revision for the fully adjusted model (BMI + Age + Sex + Indication + ASA grade).

| **BMI** | **Revision- 16.6y** | | |
| --- | --- | --- | --- |
|  | **Hazard ratio** | **LCI** | **UCI** |
| 18.5 | 1.072158 | 1.000416 | 1.149045 |
| 25 | 0.932875 | 0.870617 | 0.999585 |
| 30 | 0.838298 | 0.703484 | 0.998948 |
| 35 | 0.75277 | 0.567625 | 0.998307 |
| 40 | 0.676937 | 0.459311 | 0.997674 |

Charlson Comorbidity Index by BMI


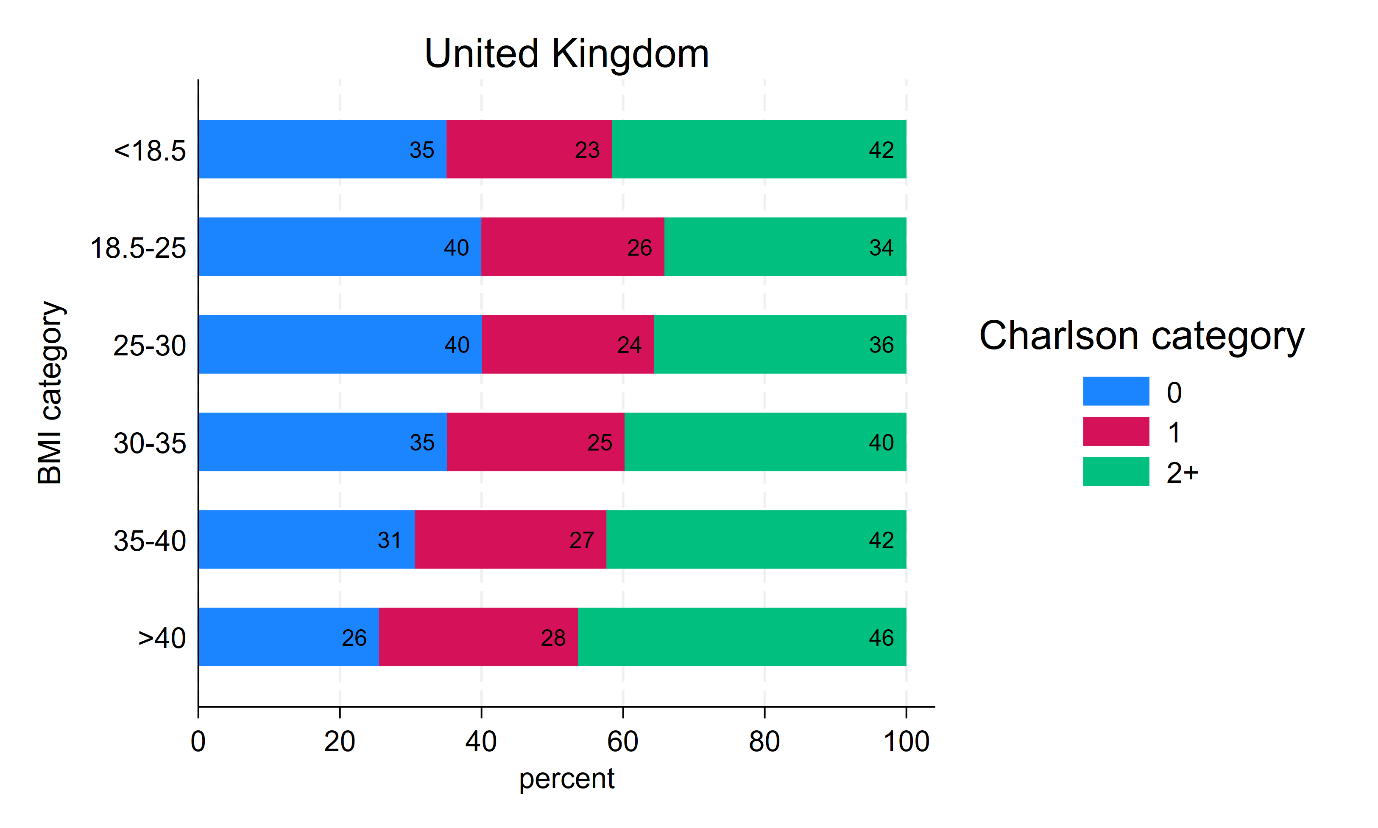


The above bar chart demonstrates the categorisation of Charlson Comorbidity Index per BMI category for the entire study population with non-missing BMI data using the linked NJR-NHS Hospital Episode Statistics dataset.


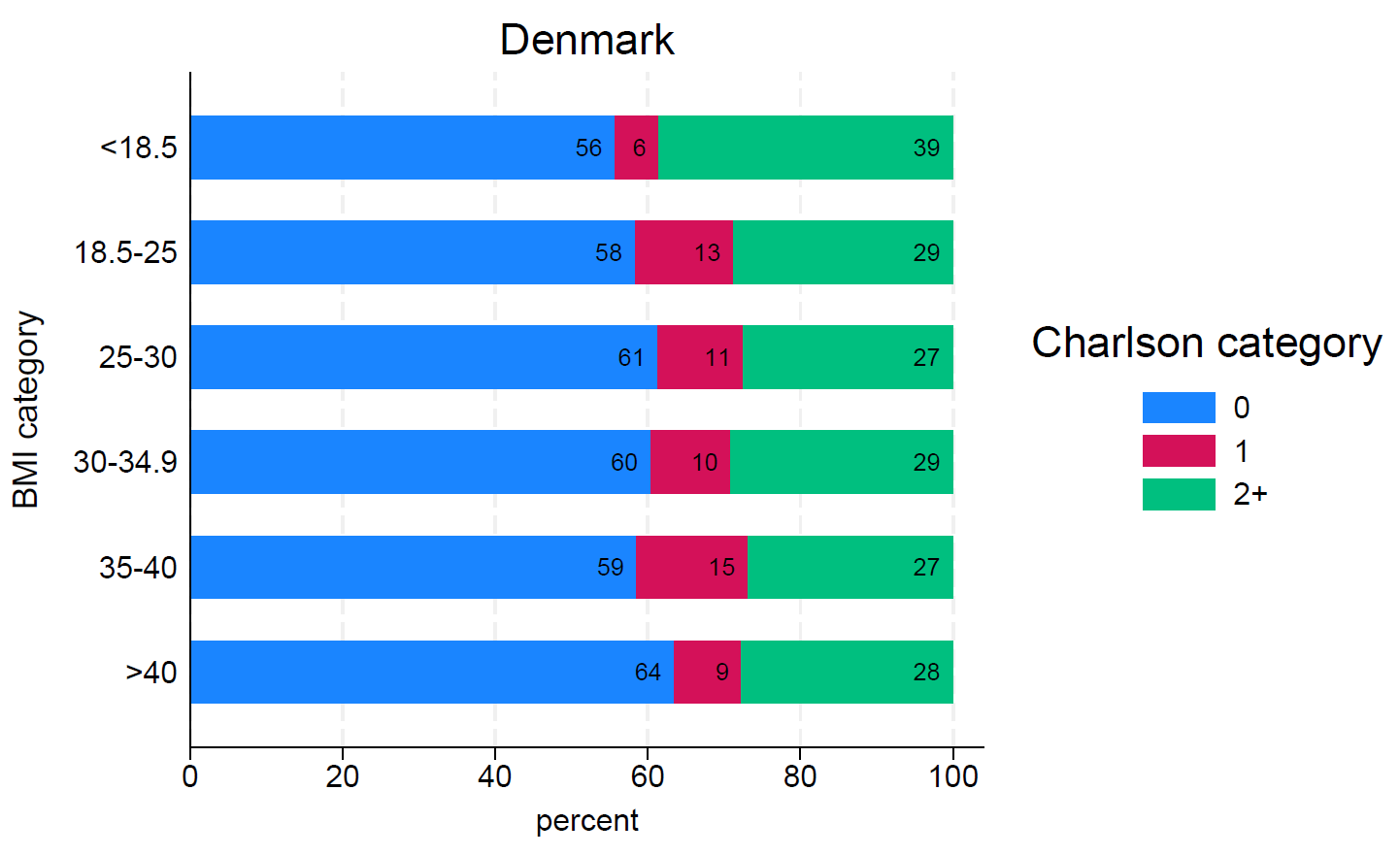

Supplement: S1 Appendix — ICD-10 codes, Data flowcharts, Incremental model and sensitivity model results, Charlson Comorbidity Index by BMI. (DOCX) [file pmed.1004786.s001.docx]
